# Cellulosic Biomass Pretreatment and Sugar Yields as a Function of Biomass Particle Size

**DOI:** 10.1371/journal.pone.0100836

**Published:** 2014-06-27

**Authors:** Michael J. Dougherty, Huu M. Tran, Vitalie Stavila, Bernhard Knierim, Anthe George, Manfred Auer, Paul D. Adams, Masood Z. Hadi

**Affiliations:** 1 The Joint BioEnergy Institute, Emeryville, California, United States of America; 2 Sandia National Laboratories, Livermore, California, United States of America; 3 Physical Biosciences Division, Lawrence Berkeley National Laboratory, Berkeley, California, United States of America; 4 Department of Bioengineering, University of California, Berkeley, California, United States of America; Queen's University Belfast, United Kingdom

## Abstract

Three lignocellulosic pretreatment techniques (ammonia fiber expansion, dilute acid and ionic liquid) are compared with respect to saccharification efficiency, particle size and biomass composition. In particular, the effects of switchgrass particle size (32–200) on each pretreatment regime are examined. Physical properties of untreated and pretreated samples are characterized using crystallinity, surface accessibility measurements and scanning electron microscopy (SEM) imaging. At every particle size tested, ionic liquid (IL) pretreatment results in greater cell wall disruption, reduced crystallinity, increased accessible surface area, and higher saccharification efficiencies compared with dilute acid and AFEX pretreatments. The advantages of using IL pretreatment are greatest at larger particle sizes (>75 µm).

## Introduction

Lignocellulosic biomass from dedicated bioenergy crops and agricultural waste has significant potential as a next generation biorefinery feedstock for the production of fuels and high value chemicals products [1]. Recalcitrance of biomass to degradation is currently a significant technical hurdle resulting in increased costs, and currently viewed as a major challenge for the scientific community [2]. Harnessing the full potential of lignocellulose will require the development of efficient pretreatment processes to enable optimal enzymatic saccharification and fermentation.

The current chemical pretreatment techniques e.g. dilute acid, ammonia fiber expansion (AFEX), and ionic liquid each have various advantages and disadvantages [1], [3]. Ionic liquid approaches when coupled with improved downstream enzyme hydrolysis have shown great promise resulting in high sugar yields from a variety of biomass sources compared to competing technologies [4], [5]. The current high cost of ionic liquids prevents this technology from being commercially viable. However, recently the economics of ionic liquid pretreatment have been recently examined and shown to depend strongly on the costs of the ionic liquid, recycling during operations and the ionic liquid to biomass loading [6].

Previous studies have reported significant differences in saccharification efficiency as a function of biomass particle size [7]–[9]. We undertook a systematic study to examine three different pretreatment regimes in order to determine the effects of particle size and biomass composition on process efficiency. We specifically examined the relative contributions of particle size and accessible surface area as a function of final sugar yield from the cellulosic biomass.

## Materials and Methods

### 2.1.1. Materials and Preparation

All switchgrass used in this study was supplied by Dr. Daniel Putman (University of California – Davis). AFEX-pretreatment was performed in the laboratory of Dr. Bruce Dale (Michigan State University, East Lansing, MI) as previously described [10]. Pretreated and untreated materials were milled with a Thomas-Wiley Mini Mill using a 40-mesh screen (Model 3383-L10, Arthur H. Thomas Co., Philadelphia, PA). The resulting material was then sifted through various screens 32–50, 75–100 and >200 µm and each particle fraction stored in a dessicator until used. Chemical composition of untreated and pretreated switchgrass samples was determined using standard NREL analytical procedure [11]. Cellulase (NS50013) and β-glucosidase (NS50010) were provided by Novozymes (Davis, CA). All other chemicals were the highest grade available and purchase from Sigma-Aldrich (St. Louis, MO).

### 2.1.2. Dilute Acid Pretreatment

Switchgrass (32–50, 75–100 and >200 µm) was dried in a convection oven at 40°C, for 24 hours, following which dried material was soaked in dilute sulphuric acid to obtain a final solids loading of 10%. After 4 hours of incubation in the acid, the mixture was loaded in a 400 mL Parr reactor (Moline, IL) and heated to 160°C, (which oscillated +/− 5°C) under a nitrogen pressure of 80 psi, to ensure constant acid concentration. The temperature was held for 20 minutes at 100 rpm stir rate, following which the reactor was rapidly cooled to 50°C in <2 minutes using rapid internal (water) and external (ice) cooling.

### 2.1.3. Ionic liquid Pretreatment

Moisture was removed from the biomass by drying to constant weight in a convection oven at 40°C. A 5% loading of biomass to ionic liquid was used (10 g biomass to 190 g [C_2_mim][OAc]). The biomass and IL slurry were placed in a 400 mL borosilicate vessel placed inside of a Parr Instrument Company Mini Bench Top Reactor (Part no. 4562). The reactor was purged of air using N_2_ and the reactants heated to 120°C while stirring at 100 rpm with venting. At temperature the reaction vessel was sealed and allowed to proceed for 3 hours. Three parts H_2_O to one part reactant was then added to the cooled reactant whilst stirring vigorously, causing precipitation of the biomass. The precipitated components were filtered using mira-cloth for subsequent processing.

### 2.2. Enzymatic Saccharification

All enzymatic saccharification reactions contained 4.1 g glucan per liter in 50 mM sodium acetate buffer, pH 5.5. in a final volume of 0.2 mL. Enzyme concentrations were normalized to 50 mg/g glucan for cellulase and 5 mg/g glucan for β-glucosidase and the reaction was incubated at 50°C in a reciprocating shaker (1000 rpm) for 20 hrs. Reducing sugar release was measured using the established DNS assay [12].

### 2.3. Crystallinity Measurement

XRD data were collected with a PANalytical Empyrean X-ray diffractometer equipped with a PIXcel^3D^ detector and operated at 45 kV and 40 kA using Cu *Kα* radiation (λ =  1.5418 Å). The patterns were collected in the 2θ range of 5 to 55°C, the step size was 0.026° with an exposure time of 300 sec. A reflection-transmission spinner was used as a sample holder and the spinning rate was set at 8 rpm throughout the experiment. The crystallinity index (CrI) was determined from the relative ratio of crystalline and amorphous peak areas by a curve fitting procedure of the measured diffraction patterns using the software package HighScore Plus® [4].

### 2.4. Scanning Electron Microscopy (SEM)

SEM images were obtained on a Hitachi S-5000 microscope for untreated and pretreated switchgrass samples as previously described [4].

### 2.5. Nitrogen Porosimetry

Nitrogen adsorption/desorption isotherms were measured using a Micromeritics ASAP 2020 surface area and porosimetry analyzer (Norcross, GA). Adsorption/desorption experiments were carried out at the temperature of liquid nitrogen (77.3 K). Adsorbed material from the surface was removed by first heating the sample tube under vacuum at a temperature of 40°C for 16 hrs. The quantity of absorbed/desorbed gas was calculated from the pressure changes using calibrated transducers. The specific surface areas were obtained by applying Brunauer, Emmett, and Teller theory [13] to nitrogen adsorption isotherms measured at liquid nitrogen temperature.

## Results and Discussion

### 3.1. Compositional Analysis

The results of composition analysis are summarized in Table 1. AFEX and IL pretreated switchgrass is similar in composition to the untreated material. Most of the xylan fraction is lost during dilute acid pretreatment, due to conversion of the hemicelluloses to sugar monomers and soluble oligomers; this sample has correspondingly higher proportions of lignin and cellulose remaining after treatment. The increased proportion of lignin in the sample may be significant, as it has been shown that lignin can negatively affect glycoside hydrolase activity [14]. Recent work has shown that both lignin content and lignin composition (*e.g.* the ratio of syringyl and guaiacyl units) can affect the efficiency of enzymatic hydrolysis [15].

**Table 1:** Compositional analysis of untreated and pretreated switchgrass.

Component	Untreated (%)	Dilute Acid (%)	AFEX (%)	Ionic liquid (%)
glucan	35.13±2.50	55.77±5.94	34.12±3.53	38.33±1.69
xylan	20.05±0.66	7.79±0.88	20.84±2.27	21.41±0.84
lignin	21.15±2.12	31.51±0.01	20.04±0.23	18.50±0.04

Compositional analysis was performed on milled biomass prior to fractionation. Data shown are a representation of three independent measurements (see Material and Methods).

### 3.2. Biomass Crystallinity

Crystallinity of lignocellulosic feedstocks has been shown to be an important determinant of enzymatic saccharification efficiency [16], [17]. Untreated and pretreated switchgrass samples were examined using powder X-ray diffraction (XRD), and the crystallinity index for each sample was calculated from the XRD pattern (Table 2). The results of this analysis shows that crystallinity is essentially unchanged by dilute acid pretreatment when compared with no pretreatment (CrI  =  0.41–0.48 versus CrI = 0.34–0.51 for untreated switchgrass) and is modestly reduced for AFEX (CrI  =  0.3–0.36). However, ionic liquid pretreated samples have significantly reduced crystallinity (CrI  =  0.15–0.2). The reduction in crystallinity is indicative of greater disruption of inter- and intra-chain hydrogen bonds in cellulose fibrils takes place, which results in greater surface area accessibility of the cellulose to enzymes. The main diffraction peaks for untreated switchgrass are located at 2θ around 16.1° and 22.3°, which correspond to the 10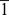 and 002 crystal lattices of cellulose I. In diluted acid treated samples, the position of the two main reflections remain essentially unchanged (16.0° and 22.1° 2θ). In contrast, the XRD patterns of the AFEX and IL-treated samples display significant difference in the peak positions and their intensity, suggesting important structural changes. Both AFEX- and IL-treated sample results in cellulose I structure disruption, with the main peak centered around 21.7° and 21.4°, respectively. The observed patterns could be due to the presence of cellulose II or distorted version of cellulose I, as indicated by the peak at around 16° 2θ [18], [19]. The broad features in the XRD patterns of the IL-treated samples are also indicative of significant amorphization of the structure occurred [20].

**Table 2:** Crystallinity index derived from XRD pattern.

Fraction (mesh)	Untreated	Dilute acid	AFEX	Ionic liquid
**32–50**	0.34	0.41	0.30	0.15
**75–100**	0.41	0.44	0.30	0.18
**>200**	0.51	0.48	0.36	0.20

Data shown are a representation of three independent measurements (see Material and Methods).

### 3.3. Scanning Electron Microscopy

**Figure 1:**
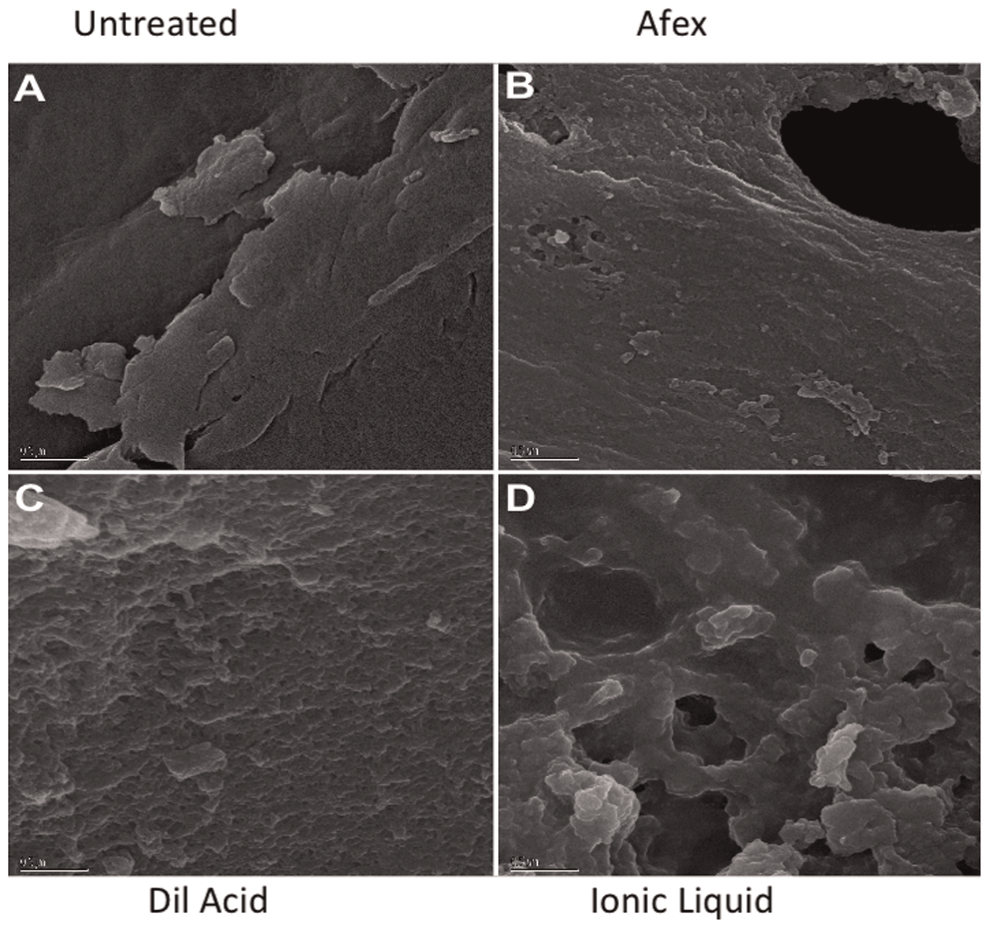
SEM images of untreated and pretreated switchgrass. A –untreated, B – AFEX-pretreated, C – dilute acid pretreated, D – ionic liquid pretreated.

SEM images of untreated and pretreated switchgrass taken at 5000x magnification clearly show the variable effects of different pretreatments on switchgrass (Fig. 1). Ionic liquid pretreatment shows greater disruption of cell wall structure than any other pretreatment, resulting in much higher surface area consistent with our data as well as previous studies [4].

### 3.4. Nitrogen Porosimetry

**Figure 2:**
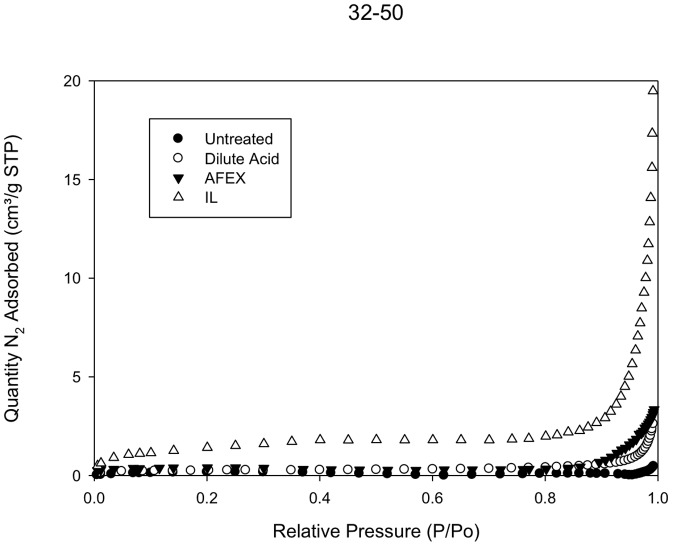
Representative plot for nitrogen porosimetry experiments. Nitrogen adsorption isotherms are shown for 32–50 mesh samples of untreated and pretreated switchgrass.

The Brunauer-Emmett-Teller (BET) surface area measurements revealed that biomass pretreatment with diluted acid, ammonia fiber expansion and ionic liquid results in more porous materials compared to untreated material. The porosimetry results are summarized in Table 3. In general, the calculated BET surface areas follow the following trend Untreated<Dilute acid<AFEX<IL. A comparison of the nitrogen adsorption isotherms for the 32–50 mesh separated samples is presented in Figure 2. The IL treated samples displays the highest amount of nitrogen adsorbed, followed by AFEX and diluted acid-treated samples. The untreated sample displays the smallest amount of nitrogen adsorbed. This is consistent with the higher BET specific area of the IL-treated sample, which is more than 6 times larger than the BET surface area of the untreated material. This is consistent with prior results on pretreatment of switchgrass using 1-ethyl-3-methyl imidazolium acetate [21]. The BET surface areas of the diluted acid and AFEX-treated biomass samples are larger by a factor of 1.5 and 2 compared to untreated biomass.

**Table 3:** BET surface areas of the biomass samples (m^2^/g).

Fraction (mesh)	Untreated	Dil. Acid	AFEX	IL
32–50	0.52	0.84	1.03	3.19
75–100	0.56	0.76	0.96	2.82
200	0.29	0.65	0.99	2.56

Data shown are a representation of three independent measurements (see Material and Methods).

### 3.5. Comparison of Saccharification Efficiency for Different Pretreatments

**Figure 3:**
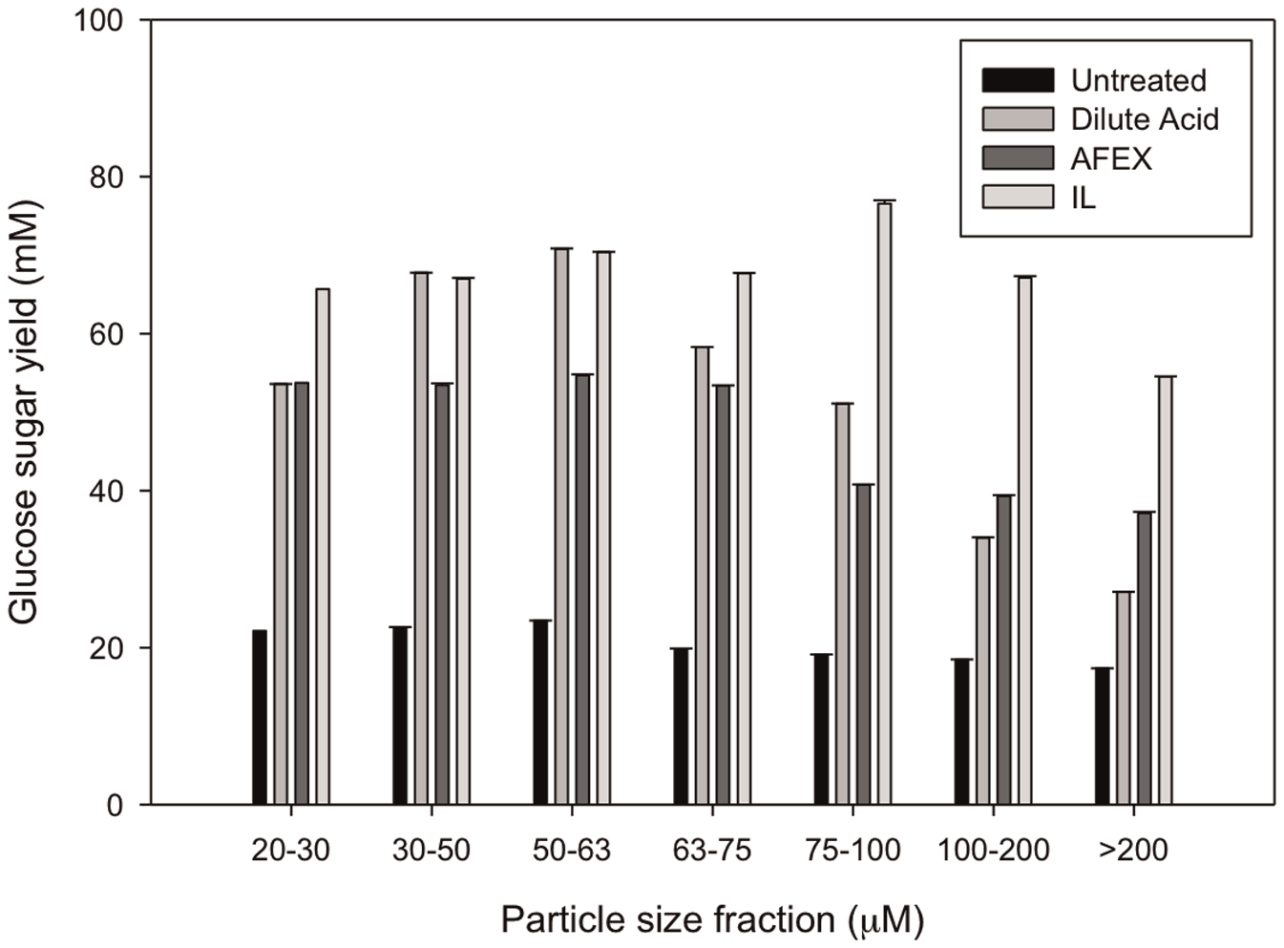
The yield of reducing sugar after enzymatic hydrolysis is shown for different particle size fractions for each pretreatment regime.

Enzymatic saccharification experiments were performed using different size fractions of untreated and pretreated switchgrass. The percentage of total glucan converted to reducing sugar for these samples is shown in Figure 3 (also Table S1). In all particle sizes, ionic liquid pretreated material shows the highest saccharification efficiency, followed by AFEX, dilute acid, and untreated samples. The effects of ionic liquid pretreatment on enzyme hydrolysis become more pronounced at larger particle sizes above 75 µm.

## Conclusions

Consistent with previous studies, ionic liquid pretreatment reduces biomass crystallinity and disrupts cell wall structure of switchgrass to a greater extent than dilute acid and AFEX pretreatments. In addition, nitrogen porosimetry experiments demonstrate that ionic liquid pretreatment results in larger amounts of accessible surface area. Finally, ionic liquid pretreatment results in higher saccharification efficiencies at every particle size tested, though the advantage over dilute acid and AFEX are greatest at larger particle sizes above 75 µm.

## Supporting Information

Table S1 Reducing sugar yields after enzymatic saccharification. Data shown are a representation of five independent measurements (see Material and Methods).
(DOCX)
