# Supplementary material for: Cellulosic Biomass Pretreatment and Sugar Yields as a Function of Biomass Particle Size
Source: PLoS One. 2014 Jun 27;9(6):e100836. doi: 10.1371/journal.pone.0100836 (PMC4074075; doi:10.1371/journal.pone.0100836)
Supplement: Table S1 — Reducing sugar yields after enzymatic saccharification. Data shown are a representation of five independent measurements (see Material and Methods). (DOCX) [file pone.0100836.s001.docx]

Supplemental Data:

Table 1. Reducing sugar yields after enzymatic saccharification. Data shown are a representation of five independent measurements (see Material and Methods).

| Fraction (Mesh) | Untreated | Dilute Acid | AFEX | Ionic liquid |
| --- | --- | --- | --- | --- |
| 20-32 | 22.1±0 | 53.5±0.2 | 53.0±0 | 65.0±0 |
| 32-50 | 22.6±0.5 | 67.7±1.2 | 53.4±5.4 | 65.2±5.4 |
| 50-63 | 22.6±3.9 | 70.7±3.5 | 54.6±3.9 | 70.0±3.4 |
| 63-75 | 19.8±2.1 | 58.2±3.5 | 53.3±1.0 | 66.7±0.8 |
| 75-100 | 19.2±1.7 | 51.0±2.0 | 40.7±0.6 | 68.9±0.2 |
| 100-200 | 18.0±0.5 | 33.0±2.0 | 39.0±2.9 | 70.1±1.8 |
| >200 | 17.3±1.9 | 27.9±1.4 | 37.1±3 | 55.9±0.4 |
